# Dialectical Thinking Is Linked With Smaller Left Nucleus Accumbens and Right Amygdala

**DOI:** 10.3389/fpsyg.2022.760489

**Published:** 2022-02-10

**Authors:** Hui-Xian Li, Xiaomeng Hu

**Affiliations:** ^1^CAS Key Laboratory of Behavioral Science, Institute of Psychology, Beijing, China; ^2^Department of Psychology, University of Chinese Academy of Sciences, Beijing, China; ^3^Department of Psychology, Renmin University of China, Beijing, China

**Keywords:** dialectical thinking, holistic thinking, reinforcement sensitivity theory, amygdala, nucleus accumbens, resting-state functional connectivity

## Abstract

Our current work examined the interface between thinking style and emotional experience at both the behavioral and neuropsychological levels. Thirty-nine Chinese participants completed the triad task, and we calculated the rate of individually selected relationship pairings to overall selections to represent their holistic thinking tendencies. In addition, participants in the top one-third of the ratio score were classified into the high holistic thinking group, while those in the bottom one-third of the ratio score were classified into the low holistic thinking group. We used the sensitivity to punishment and sensitivity to reward questionnaire (SPSRQ) to examine how people elicit positive and negative affective behaviors. Additionally, we examined the volume of the amygdala and nucleus accumbens and their functional connectivity in the resting-state. We found that high holistic thinkers were much less sensitive to rewards than low holistic thinkers. In other words, individuals with high holistic thinking are less likely to pursue behaviors that have positive emotional outcomes. Furthermore, their bilateral nucleus accumbens and right amygdala volumes were smaller than those of low holistic thinkers. Hierarchical regression analysis showed that holistic thinking tendency can negatively predict the volume of the left nucleus accumbens and right amygdala. Finally, resting-state functional connectivity results showed increased functional connectivity FC between left nucleus accumbens and bilateral amygdala in high holistic thinkers. These findings provide emotion-related manifestations of thinking styles at the behavioral and neural levels.

## Introduction

Peng et al. (2006) divided Chinese dialectical epistemology into three interrelated principles to facilitate empirical analysis of dialecticism. First, the principle of change holds that reality is a dynamic and flexible process, and people, events, and experiences can change into opposites (e.g., positive to negative). Second, the principle of contradiction asserts that reality is complex and full of contradiction (“dividing one into two”). Finally, the principle of holism maintains that nothing in the universe is isolated and independent, and everything is connected and relational. They proposed that the principle of change leads to a belief in contradiction that results from a belief in change. Thus, holism is a consequence of the principles of change and contradiction. The Chinese holistic thought pattern is focused on two basic assumptions of Taoism: two poles (yin and yang) and five elements (metal, wood, water, fire, and earth). A straightforward understanding of holism is that even simple events depend on a variety of complex relationships. What we need to note is dialecticism is a distinct construct from collectivism and interdependence (Wong and Liu, 2018).

A growing body of work has illuminated that East Asians think holistically, while Westerners think analytically in terms of attention, perception, and cognition (Nisbett and Masuda, 2003; Nisbett and Miyamoto, 2005; Spencer-Rodgers and Peng, 2018). Holistic thinking involves understanding a system by sensing its larger-scale patterns and giving broader attention to the context, relationships, and background elements. Analytical thinking involves understanding a system by thinking about its parts and how they work together to produce large-scale effects as well as having a narrow focus on objects in the foreground and tending to disentangle phenomena from the contexts in which they are embedded. The holistic thinkers attend to the entire field, assigning causality to it, making relatively little use of categories and formal logic, and relying on “dialectical” reasoning (Nisbett et al., 2001). Dialectical thinking influences how people evaluate themselves, their lives, and their subjective wellbeing. Cross-cultural research shows that East Asians report less positive affect, lower life satisfaction, and lower subjective wellbeing than Westerners (Lee and Seligman, 1997; Kitayama et al., 2000; Spencer-Rodgers et al., 2004; Lee and Wu, 2008; Cheng et al., 2011; Wong et al., 2011).

Although most researchers have demonstrated that dialecticism is correlated with lower subjective wellbeing (Spencer-Rodgers et al., 2004; Hamamura et al., 2008; Chen et al., 2013), it is unclear whether dialecticism has a positive or negative impact on mental health (Wong and Liu, 2018). It is worth noting that the dominant concept of subjective wellbeing is to obtain a global wellbeing score by subtracting negative emotional scores from positive emotional scores (Schimmack, 2007). This calculation method reflects European and American cultures. Researchers have found that individuals’ lay beliefs about subjective wellbeing shape their experience of certain aspects of subjective wellbeing; people with dialectical beliefs about subjective wellbeing experience lower levels of positive affect because positive affect is a less relevant consideration in their conceptualization of subjective wellbeing (Wong and Liu, 2018). Holistic thinkers embrace the changing nature of happiness and coexistence of positive and negative emotions, maintain a balance between moderate emotions, and prefer to aim for emotional moderation (Leu et al., 2011). The dialectical thinker refrains from pursuing extreme positive emotions (Miyamoto and Ryff, 2011) on the one hand, and balances positive emotions by accepting negative emotions on the other hand, which may lead to lower subjective wellbeing scores according to current definitions and calculations. In addition, concepts related to subjective wellbeing, such as self-esteem, were also measured by subtracting negativity from positivity; negatively worded items on the self-esteem scale were scored inversely so that all items were summed to form an overall assessment of self-esteem (Wong and Liu, 2018). However, dialectical cultures accept the coexistence of good and bad in their lives and embrace contradictory or dichotomies of self-evaluations (Spencer-Rodgers et al., 2004; Hamamura et al., 2008; Boucher et al., 2009); negative evaluations of the self are not necessarily equivalent to the absence of positive self-evaluations (Wong and Liu, 2018).

Therefore, researchers have suggested measuring positive and negative affect separately (Schimmack, 2007; Wong and Liu, 2018). To gain insight into the behavioral responses and neural mechanisms of the brain related to emotional processing in individuals with different thinking styles, the current study explored both behavioral and neural substrates. Specifically, we tested the extent to which thinking styles tended to elicit positive and negative affective behaviors. We then explored the effect of thinking styles on the structure and function of brain regions associated with emotions.

Gray’s reinforcement sensitivity theory (RST) (Gray, 1970; Gray and McNaughton, 2000; McNaughton and Corr, 2004), a prominent neuroscience theory of personality, consists of three major brain systems that regulate the intensity of approach and withdrawal behavior in response to emotional stimuli: the behavioral inhibition system (BIS), behavioral activation system (BAS), and fight-flight-freeze system (FFFS). The BAS is responsible for approaching behavior in response to pleasant stimuli along with positive emotional experiences. The BIS controls behavior in response to goal conflict. The BIS is activated when a goal conflict stimulus is presented and accompanied by anxiety, which inhibits otherwise dominant behavior in the conflict and seeks the best way to resolve the conflict. The FFFS system is activated by all conditioned and unconditioned aversive stimuli that regulate defensive avoidance behavior along with negative emotional experiences (fear). These systems reflect the brain structures that influence sensitivity to reinforcing events and control emotional experiences (Torrubia et al., 2001). The RST shows the existence of two general traits (Adrián-Ventura et al., 2019) that can be assessed using self-report questionnaires. The first is sensitivity to punishment (SP), which reflects the responsiveness of the FFFS and BIS, and the second is sensitivity to reward (SR), which reflects the responsiveness of the BAS (Torrubia et al., 2001). Furthermore, the previously studied BIS reflects the combined BIS and FFFS functions (Corr, 2004). Individuals with high SR/BAS exhibit more approach behavior to achieve positive emotion reinforcement, whereas individuals with high SP/BIS exhibit more behavioral inhibition to avoid negative emotion (Smillie et al., 2006). Thus, the RST can provide meaningful information for understanding individuals’ behavioral responses to pursue positive emotional experiences and avoid negative emotional experiences. Here, we investigate whether there were differences in the behavioral reflective traits of individuals related to emotional experiences under the influence of their thinking style. In other words, does an individual’s way of thinking influence their performance of behaviors related to emotional experiences? As mentioned above, holistic thinkers’ dialectical beliefs about subjective wellbeing include moderation and balance in their affect, such as avoiding strong affect (e.g., extreme joy begets sorrow) and desires (e.g., content with what you have not greedy) (Wong et al., 2011). Thus, we believe that people with high holistic thinking tendencies are less likely to engage in approach behaviors to obtain positive emotions than people with low holistic thinking tendencies (i.e., lower SR scores). Meanwhile, we believe that high holistic thinkers tend to avoid negative emotional experiences; thus, they have higher SP scores.

The RST is a biologically based model; therefore, it can be combined with neurophysiology. In Gray (1987) researchers discovered that the activation of the BAS was associated with the activity of the midbrain limbic dopamine pathway concentrated in the nucleus accumbens. The responsiveness of the BIS/BAS system depends on environmental inputs, whereas the sensitivity of the system is biologically based (Scholten et al., 2006). In other words, self-report questionnaires measure an individual’s beliefs about responsiveness to a stimulus, whereas biometric measures are direct indicators of individual sensitivity. Neurobiological factors (e.g., brain structure and function) play a vital role in our understanding of different thinking styles. Of particular interest, the nucleus accumbens and amygdala are seen as an intuitive emotional system (Haruno et al., 2014), and their structural alterations have been known to be associated with psychopathology (Tottenham and Galván, 2016). The nucleus accumbens is a key node of the reward circuit, plays a key role in reward-related approach and avoidance behavior, and links motivation and emotion to regulated action (Haber and Knutson, 2010; Humphries and Prescott, 2010; Namburi et al., 2015; Piantadosi et al., 2017). Furthermore, the amygdala plays an important role in emotional processing as a point of divergence in the circuitry that mediates positive and negative emotions or motivational values, with a large number of fiber projections to the nucleus accumbens (Haber and Knutson, 2010; Namburi et al., 2015; Piantadosi et al., 2017). Studies in mice have demonstrated a causal relationship between the activity of bilateral amygdala neurons projecting to the nucleus accumbens and reward-related behaviors and between bilateral amygdala neurons projecting to the centromedial amygdala and negative reinforcement (avoidance) (Namburi et al., 2015).

Furthermore, cultural neuroscience has demonstrated that culture influences the anatomical or functional features of the brain (Kitayama and Uskul, 2011). For example, Kitayama et al. (2017) found that the gray matter (GM) volume of the bilateral orbitofrontal cortex can predict interdependent self-construal. Wang et al. (2017) found that independence was associated with increased GM volume in many self-related regions such as the right rostrolateral prefrontal cortex. A meta-analysis of cultural differences in human brain activity suggests that cultural differences in social and non-social processes are mediated by distinct neural networks such as the anterior cingulate and bilateral frontal cortex (Han and Ma, 2014). Bacha-Trams et al. (2018) found that holistic and analytical individuals exhibited different brain activities when watching the same drama movie. Furthermore, researchers have identified significant cultural differences in emotional processing tasks in areas such as the dorsolateral prefrontal cortex, temporal-parietal junction, median insula, and subcortical areas (De Greck et al., 2012; Park et al., 2016, 2018). For example, Park et al. (2016, 2018) found that ventral striatal activity mediates cultural differences in affiliative judgments of smiles. Culture influences the neural correlates of emotional processing and, thus, the amygdala response (Phelps et al., 2000; Cunningham et al., 2004; Chiao et al., 2008; Derntl et al., 2009, 2012). For example, Chiao et al. (2008) found that the bilateral amygdala response to fearful faces was modulated by culture. Specifically, the responsivity of the amygdala increases when fear faces are detected in members of one’s group relative to other cultural groups. A recent study demonstrated that resting-state brain network properties (graph theory) can reflect an individual’s holistic analytic thinking style. In particular, they found that functional graph metrics of the basal ganglia and amygdala are important predictors for distinguishing individual thinking styles (Luo et al., 2021). Therefore, the amygdala and nucleus accumbens can reflect individuals with different thinking styles’ behavioral responsiveness to positive and negative emotions.

Therefore, our study aimed to investigate the relationship between thinking styles, behavioral responses to positive and negative emotions, and their neural substrates. First, we examined group differences in participants with holistic versus analytical thinking styles using a triad task (Talhelm et al., 2014). Participants selected one of two images that they thought matched the target image. One selection type belongs to the same abstract category (analytical thinking) as the target picture (e.g., chickens and cattle belong to the animal category), and the other has a functional relationship (holistic thinking) with the target picture (e.g., cattle eating grass). One disadvantage of cross-cultural research comparing analytical and holistic thinking styles is that it is difficult to control the presence of other culture-specific variables that might co-vary with analytical-holistic cognitive styles (Bacha-Trams et al., 2018). Therefore, we studied holistic and analytical participants within the Chinese naïve dialecticism culture, as there is a spectrum of individuals with analytical to holistic cognitive styles within each culture (Kitayama et al., 2006; Talhelm et al., 2014). Therefore, we calculated the ratio of the selected relational pairings to the overall selection to represent individuals’ holistic thinking tendencies.

On the one hand, we used the Sensitivity to Punishment and Sensitivity to Reward Questionnaire (SPSRQ) (Torrubia et al., 2001) to evaluate the extent and sensitivity with which individuals experience positive and negative emotions, detect approaches toward positive emotional behaviors, and avoid negative emotional behaviors. This questionnaire is suitable for assessing self-reported sensitivity to social reward and punishment, social tendencies, and avoidance behavior (Fussner et al., 2018). On the other hand, we collected the structural and resting-state functional images using magnetic resonance imaging (MRI) to examine whether structural and functional differences of the nucleus accumbens and amygdala exist between holistic and analytical thinkers. Researchers have demonstrated that resting-state functional MRI (R-fMRI) signals can effectively predict psychological tendencies and can be used to clarify different types of neurological and psychiatric diseases (Biswal et al., 1995; Zhang and Raichle, 2010; Yan et al., 2019; Luo et al., 2021). As mentioned above, projection connections between the amygdala and nucleus accumbens are strongly linked to the regulation of reward-and punishment-related behaviors. Therefore, we examined the functional connectivity (FC) between the bilateral amygdala and nucleus accumbens in the resting state, which reflects spontaneous brain activity. This is independent of the structural MRI analysis. Finally, we combined RST with neurophysiology. Specifically, we examined the relationships among thinking style, reinforcement sensitivity, and nuclei volume using mediation analysis. We assumed that people with high holistic thinking tendencies are not sensitive to reward (low approach to positive emotions), so their nucleus accumbens and amygdala volumes would be smaller.

In short, we first tested the hypothesis that holistic thinkers would be less prone to pursuing positive emotions and more avoidant of things that cause negative emotions. Then, we related thinking styles to the volume of the amygdala and nucleus accumbens and their functional connectivity in the resting state. In addition, we examined the relationships among thinking style, sensitivity to reward, and nuclei volume.

## Materials and Methods

### Participants

Participants were recruited through internet advertisements. Exclusion criteria included neurological or psychiatric disorders, use of psychotropic medication, and any history of substance or alcohol abuse. Thirty-nine (18 males; age range: 18–28 years; mean age: 21 years) young and healthy Chinese adult participants completed the MRI scanning, triad task, and questionnaire measurements. All participants were educated to undergraduate level and above. In addition, the objective average annual family income of participants was around 100,000 RMB. The mean subjective rating of the socioeconomic status of the family was 4.47 (rating range: 1–10) with a standard deviation (SD) of 1.61 (see Supplementary Table 1 for additional demographic information). Approval was obtained from the institutional review board of the Institute of Psychology, Chinese Academy of Sciences. All the participants provided written informed consent.

### Questionnaires

The SPSRQ (Torrubia et al., 2001) consists of 48 yes-no response items that contain two independent 24-item scales: sensitivity to punishment (SP) and sensitivity to reward (SR). The Chinese version of SPSRQ (SPSRQ-CV) (En-Jie, 2012) removed 12 items that were not closely related to the life of Chinese or inconsistent with their way of thinking, but were consistent with the original SPSRQ scale structure. Cronbach’s α of the SR (16-item) and SP (18-item) was 0.64 and 0.80, respectively. The test-retest reliability of the SR and SP was 0.89 and 0.61, respectively. The Cronbach’s α calculated for our sample is 0.60 and 0.81 for SR and SP, respectively.

### Evaluate the Holistic-Analytical Thinking Styles

Participants were asked to freely select one of the two images that they thought matched the target image. The selected items were of two types: One type belonged to the same abstract category (i.e., analytical thinking) as the target picture (e.g., chickens and cattle belong to the animal category), and the other had a functional relationship (i.e., holistic thinking) with the target picture (e.g., cattle eats grass). The task consisted of 14 different selection trials (see Supplementary Materials for all the stimuli).

The task-fMRI experiment obtained two types of pictures that were selected by the participants. The results showed that 39 participants chose more relational pairings (the number of relational pairings: 9.38 ± 2.84; number of category pairings: 4.13 ± 2.92; T_38_ = 5.74, *p* < 0.01). This may be due in large part to the fact that the participants of our study were Chinese. Therefore, we calculated the ratio of selected relational pairings to the overall selection. Participants in the top one-third of the ratio score were categorized into the high holistic thinker group or holistic thinking tendency group (0.90 ± 0.07). Those in the bottom one-third of the ratio score were categorized into the low holistic thinker group or the analytical tendency group (0.51 ± 0.19). The differences between the two groups were considerable (T_20.01_ = 7.64, *p* < 0.01). We also grouped participants into two groups (top half and bottom half) by the median. The results are presented in Supplementary Table 7.

### Magnetic Resonance Imaging Data Acquisition

Magnetic resonance imaging data were acquired using a GE MR750 3.0T scanner with an 8-channel cranial coil at the MRI Research Center, Institute of Psychology, Chinese Academy of Sciences. T1-weighted anatomical images were acquired using a 3D-SPGR pulse sequence [192 sagittal slices, repetition time (TR) = 6.65 ms, echo time (TE) = 2.93 ms, flip angle (FA) = 12°, field of view (FOV) = 256 mm × 256 mm, matrix size = 256 × 256, slice thickness = 1 mm, voxel size = 1 × 1 × 1 mm^3^]. The functional data were acquired with echo-planar imaging (EPI) sequence (37 axial slices, TR = 2000 ms, TE = 30 ms, FA = 90 degrees, FOV = 224 mm × 224 mm, matrix size = 64 × 64, slice thickness = 3.5 mm, voxel size = 3.5 × 3.5 × 3.5 mm^3^).

### Magnetic Resonance Imaging Data Preprocessing

The MRI data were preprocessed using DPABISurf (Yan et al., 2021) which is a surface-based R-fMRI data analysis toolbox evolved from DPABI/DPARSF. It calls fMRIPprep (Esteban et al., 2019) to preprocess the structural and functional MRI data. (1) The anatomical data preprocessing was as follows: the T1-weighted image was corrected for intensity non-uniformity (INU) with N4BiasFieldCorrection (Tustison et al., 2010), distributed with ANTs 2.2.0 (Avants et al., 2008) and used as a T1w-reference throughout the workflow. The T1w-reference was then skull-stripped with a Nipype implementation of the antsBrainExtraction.sh workflow (from ANTs) using OASIS30ANTs as the target template. Brain tissue segmentation of cerebrospinal fluid (CSF), white matter (WM), and gray matter (GM) was performed on brain-extracted T1w using fast (Zhang et al., 2001). Brain surfaces were reconstructed using recon-all (Dale et al., 1999), and the brain mask estimated previously was refined with a custom variation of the method to reconcile ANT-derived and FreeSurfer-derived segmentation of the cortical GM of Mindboggle (Klein et al., 2017). Volume-based spatial normalization to one standard space (MNI152NLin2009cAsym) was performed through non-linear registration with antsRegistration (ANTs 2.2.0) using brain-extracted versions of both T1w reference and the T1w template. The following template was selected for spatial normalization: ICBM 152 non-linear asymmetrical template version 2009c (Fonov et al., 2009). (2) Functional data preprocessing was performed as follows. First, a reference volume and its skull-stripped version were generated using a custom methodology of fMRIPrep. The blood oxygen level-dependent (BOLD) reference was then co-registered to the T1w reference using the bbregister (FreeSurfer), which implements boundary-based registration (Greve and Fischl, 2009). The BOLD run was slice-time-corrected using 3dTshift (Cox and Hyde, 1997). The BOLD time-series were resampled into standard space, generating a preprocessed BOLD run in the “MNI152NLin2009cAsym” space. Nuisance regression of the resting-state data was performed as follows: The Friston 24-parameter model (Friston et al., 1996) was used to regress out head motion confounds. Other sources of spurious variance (WM and CSF signals) were also removed from the data using linear regression to reduce respiratory and cardiac effects. Furthermore, linear trends were included as regressors to account for drifts in the BOLD signal. Finally, a bandpass temporal filter (0.01–0.1 Hz) and spatial smoothing (full width at half maximum of 6 mm) were applied to the normalized functional images.

### Volumetric Acquisition of the Nucleus Accumbens and Amygdala

The file (ResultsS-AnatVolu: Anat_Segment_Volume.tsv) after DPABISurf preprocessing yielded the clump sizes of all subjects generated by Freesurfer. We extracted the volumes of the bilateral nucleus accumbens and amygdala.

### Functional Connectivity Analysis of the Resting State

We defined each participant’s bilateral amygdala and nucleus accumbens as anatomical regions of interest (ROIs) based on the “MNI152NLin2009cAsym” space. We extracted the average time series from each ROI and calculated the FC between each ROI pair using Pearson’s correlation. Fisher’s r-to-z was then used to transform all *R* to *Z*-values.

### Mediation Analysis

We used the PROCESS tool in SPSS (Hayes, 2017) to test the mediation of SR on nucleus accumbens volume and SP on amygdala volume. The bootstrap approach was used to test significance with 5000 bootstrap resampling to generate a 95% confidence interval.

## Results

### Group Differences in Sensitivity to Reward and Punishment

**FIGURE 1:**
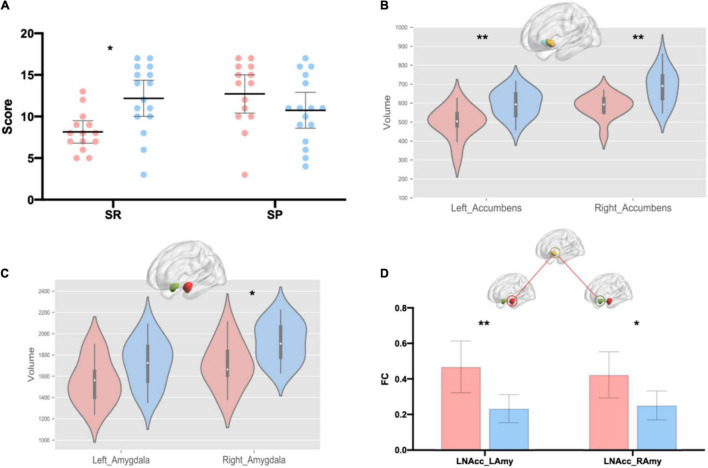
Group differences in the SPSRQ, volume of nucleus accumbens and amygdala, and resting-state functional connectivity between the bilateral amygdala and left nucleus accumbens. **(A)** Group differences in the sensitivity to reward and punishment. Data are means with 95% CI. **(B)** Group differences in bilateral nucleus accumbens. **(C)** Group differences in the bilateral amygdala. **(D)** Group differences in the resting-state functional connectivity between the bilateral amygdala and left nucleus accumbens. Pink represents the high holistic thinker group and blue represents the low holistic thinker group. SR, sensitivity to reward, SP, sensitivity to punishment, LNAcc, left nucleus accumbens; LAmy, left amygdala; RAmy, right amygdala; FC, functional connectivity. *p < 0.05; **p < 0.01.

First, we tested the normality of these variables (Supplementary Table 2). Spearman’s rank correlation was used to calculate the correlation for data involving non-normal distributions; otherwise, Pearson’s correlation was used. Holistic thinking tendency was negatively correlated with SR [Spearman’s rho (39) = –0.37, *p* = 0.02] but was not correlated with SP [Spearman’s rho (39) = 0.20, *p* = 0.23] (Table 1). Furthermore, we found that high holistic thinkers were much less sensitive to rewards than were low holistic thinkers [Figure 1A; T_28_ = –2.47, *p* = 0.02, Cohen’s d = 0.90, 95% CI = (–3.86, –0.36)], while their SP was not different from that of low holistic thinkers [Figure 1A; T_28_ = 1.34, *p* = 0.19, Cohen’s d = 0.49, 95% CI = (–1.05, 4.98)].

**TABLE 1:** The correlation between holistic thinking tendency, sensitivity to reward and punishment, the volume of bilateral nucleus accumbens, and amygdala (*n* = 39).

	Mean	SD	HT^#^	SR^#^	SP^#^	LNAcc	RNAcc	LAmy
Sensitivity to reward (SR)	9.44	2.64	–0.37*					
Sensitivity to punishment (SP)	11.36	4.10	0.20	–0.21				
the volume of								
Left nucleus accumbens (LNAcc)	553.13	82.51	–0.52**	0.38*	–0.07			
Right nucleus accumbens (RNAcc)	629.45	87.48	–0.46**	0.31^a^	–0.15	0.64**		
Left amygdala (LAmy)	1647.72	216.32	–0.19	0.11	0.00^b^	0.42**	0.49**	
Right amygdala (RAmy)	1826.26	210.70	–0.32*	0.14	–0.01	0.49**	0.56**	0.88**

^#^Non-normal distribution. ^a^p = 0.054. Two-tailed. *p < 0.05; **p < 0.01. b:0.003.

### Nucleus Accumbens and Amygdala Volumes Compared Between the Two Groups

The results showed that holistic thinking tendency was negatively related to bilateral nucleus accumbens volume [Table 1; left: Spearman’s rho (39) = –0.52, *p* < 0.01; right: Spearman’s rho (39) = –0.46, *p* < 0.01] and right amygdala volume [Table 1; Spearman’s rho (39) = –0.32, *p* < 0.05]. Furthermore, we found that high holistic thinkers had smaller volumes in the left nucleus accumbens [Figure 1B; T_28_ = –3.61, *p* < 0.01, Cohen’s d = 1.32, 95% CI = (–155.01, –42.87)] compared to low holistic thinkers. In addition, the right nucleus accumbens volumes were smaller in the high holistic thinkers [Figure 1B; T_28_ = –3.28, *p* < 0.01, Cohen’s d = 1.20, 95% CI = (–152.16, –35.14)]. The volume of the right amygdala was smaller in high holistic thinkers than in low holistic thinkers [Figure 1C; T_28_ = –2.17, *p* = 0.04, Cohen’s d = 0.79, 95% CI = (–321.98, –9.29)]. Likewise, the left amygdala volumes were smaller in high holistic thinkers, but the difference between the two groups was not significant [Figure 1C; T_28_ = –1.61, *p* = 0.12, Cohen’s d = 0.59, 95% CI = (–285.80, 34.25)].

### Hierarchical Regression Analysis

In the present study, we have three different aspects of variables: holistic thinking tendency, SR and SP, and the volume of the nucleus accumbens and amygdala. The reasons for using hierarchical regression in this study are as follows. First, a unique contribution is detected. In addition to the relationship between holistic thinking tendency and nucleus volume, we found that SR was positively correlated with left nucleus accumbens volume [Spearman’s rho (39) = –0.38, *p* < 0.05]. The positive correlation between SR and right nucleus accumbens volume was borderline significant [Spearman’s rho (39) = –0.31, *p* = 0.05]. Second, there was a correlation between the independent variables. Our results show a significant correlation between holistic thinking tendencies and SR. The correlation results for all variables are presented in Table 1. Third, the procedure provides us with an opportunity to explore the incremental validity of the variables simultaneously by the change in *R*^2^ in the hierarchical regression analysis. In general, a hierarchical regression analysis was performed to speculate on the causal effect of holistic thinking tendency on the volume of the nucleus accumbens and amygdala and detect the degree of contribution of different variables. All thirty-nine individuals were used for the hierarchical regression analysis. The variables were entered in the following order: (i) demographic information, (ii) SR and SP, and (iii) holistic thinking tendency.

The results showed that holistic thinking tendency significantly negatively predicted the volume of the left nucleus accumbens. Furthermore, participants’ holistic thinking tendencies accounted for 13.7% of the total variance in the volume of the left nucleus accumbens (Table 2). However, holistic thinking tendency did not help predict the participants’ right nucleus accumbens volume. For the amygdala, the results revealed that both sexes (left amygdala: β = –0.60, *t* = –3.86, *p* < 0.01; right amygdala: β = –0.61, *t* = –4.40, *p* < 0.01) and age (left amygdala: β = –0.51, *t* = –2.57, *p* < 0.05; right amygdala: β = –0.57, *t* = –3.25, *p* < 0.01) had a significant impact on participants’ volume of the bilateral amygdala. Furthermore, the results revealed a negative predictive impact of holistic thinking tendency on the volume of the right amygdala, which accounted for 10.7% of the variance (Table 3). The holistic thinking tendency did not help predict the participants’ left amygdala volume. Therefore, the results revealed predictive impacts of holistic thinking tendency on the volume of the left nucleus accumbens and right amygdala when other variables were controlled.

**TABLE 2:** Hierarchical regression predicting the volume of left and right nucleus accumbens.

	Left nucleus accumbens (LNAcc)					Right nucleus accumbens (RNAcc)				
Predictors	Beta	*t*	*p*	R^2^ change	*p*	Beta	*t*	*p*	R^2^ change	*p*
Step 1				0.340	0.016				0.426	0.002
Gender	–0.21	–1.55	0.132			–0.26	–1.80	0.083		
Age	–0.29	–1.67	0.107			–0.31	–1.68	0.104		
Education	0.25	1.53	0.136			0.11	0.60	0.553		
Average annual family income	0.30	1.57	0.128			0.33	1.61	0.119		
Assessment of the socio-economic status of the family	0.01	0.06	0.950			0.13	0.75	0.459		
Step 2				0.083	0.135				0.012	0.723
Sensitivity to Reward (SR)	0.18	1.29	0.208			0.07	0.44	0.660		
Sensitivity to Punishment (SP)	0.26	1.92	0.064			0.10	0.66	0.513		
Step 3				0.137	0.005				0.061	0.070
Holistic thinking tendency (HT)	–0.43	–3.01	0.005			–0.29	–1.88	0.070		

**TABLE 3:** Hierarchical regression predicting the volume of left and right amygdala.

	Left amygdala (LAmy)					Right amygdala (RAmy)				
Predictors	Beta	*t*	*p*	R^2^ change	*p*	Beta	*t*	*p*	R^2^ change	*p*
Step 1				0.398	0.005				0.427	0.002
Gender	–0.60	–3.86	0.001			–0.61	–4.40	0.000		
Age	–0.51	–2.57	0.016			–0.57	–3.25	0.003		
Education	0.16	0.85	0.404			0.21	1.27	0.214		
Average annual family income	–0.24	–1.10	0.279			–0.16	–0.81	0.424		
Assessment of the socio-economic status of the family	0.24	1.28	0.209			0.19	1.18	0.248		
Step 2				0.004	0.915				0.019	0.606
Sensitivity to Reward (SR)	0.01	0.03	0.976			–0.04	–0.29	0.773		
Sensitivity to Punishment (SP)	0.07	0.46	0.651			0.17	1.20	0.240		
Step 3				0.037	0.177				0.107	0.013
Holistic thinking tendency (HT)	–0.23	–1.39	0.177			–0.38	–2.64	0.013		

### Resting-State Functional Connectivity Between the Bilateral Amygdala and Nucleus Accumbens

We calculated correlations between the volume of the nucleus accumbens and amygdala and FC (Supplementary Table 3). We found a significant positive correlation between the right amygdala volume and FC between the right amygdala and right nucleus accumbens (*r* = 0.35, *p* = 0.03). We compared FC strengths between high and low holistic thinkers. High holistic thinkers demonstrated increased FC between the bilateral amygdala and left nucleus accumbens compared to low holistic thinkers [Figure 1D; LNAcc_LAmy: T_28_ = 3.17, *p* < 0.01, Cohen’s d = 1.16, 95% CI = (0.08, 0.39); LNAcc_RAmy: T_28_ = 2.49, *p* < 0.05, Cohen’s d = 0.91, 95% CI = (0.03, 0.32)].

### Mediation Analysis

We tested whether SR mediates holistic thinking tendencies and the volume of the nucleus accumbens and amygdala. The results showed that SR partially mediated the relationship between thinking tendency and the volume of the left nucleus accumbens (Table 4). However, the mediating effect of SR on the right nucleus accumbens was not significant (Table 4). In contrast, we found no mediating effect of SR on the amygdala.

**TABLE 4:** Statistic on indirect and direct effects (X = holistic thinking tendency, Mediator = reward sensitivity, Y = the volume of nucleus accumbens and amygdala, *n* = 39).

Path	Effect	Boot SE	Boot LL CI 95%	Boot UL CI 95%	Effect	Boot SE	Boot LL CI 95%	Boot UL CI 95%
Left_ nucleus accumbens					Right_ nucleus accumbens			
Indirect effect	–31.29	22.84	–100.16	–0.08	–28.19	28.82	–131.53	5.34
Direct effect	–163.90	55.36	–276.18	–51.62	–154.96	61.86	–280.44	–29.48
Left_ amygdala					Right_ amygdala			
Indirect effect	–20.52	73.81	–180.05	106.05	–10.37	64.37	–146.05	109.03
Direct effect	–131.47	174.33	–485.03	222.09	–276.41	164.61	–610.27	57.45

*PROCESS model 4 was used. SE, standard error; CI, confidence interval; LL, lower limit; UL, upper limit.*

## Discussion

To the best of our knowledge, this is among the first studies to link the reinforcement sensitivity theory and neural substrates of holistic versus analytical thinking, providing behavioral and biological mechanisms to support the link between different ways of thinking.

First, we used the SPSRQ based on reinforcement sensitivity theory to evaluate individual sensitivity to positive and negative emotions. On the one hand, we found that the SR of high holistic thinkers was lower than that of low holistic thinkers; that is to say, high holistic thinkers were less likely to pursue extreme positive emotions. Different thinkers’ beliefs about mental health may partially explain our results. Tsai et al. (2006) showed that Hong Kong Chinese value high-arousal positive affect less than European Americans and value low-arousal positive affect more than European Americans. The values people place on things influence their behavior, and they tend to promote their mental health by looking for experiences that best fit their beliefs (Wong and Liu, 2018). Wong et al. (2011) also demonstrated that participants who endorsed dialectical beliefs reported less positive affect. Our behavioral results revealed less behavioral approach behavior toward positive emotions in individuals with a high holistic thinking tendency. Therefore, holistic beliefs regarding mental health that emphasize moderation may result in lower levels of high-arousal positive affect and approach behavior given that the pursuit of positive affect is less relevant to these beliefs. On the other hand, we did not find a significant difference between the high and low groups in terms of SP, suggesting that there was no difference between individuals with high and low holistic thinking tendencies in terms of avoiding things that bring negative emotions. Furthermore, we did not find a correlation between SP and holistic thinking tendency (Table 1). These results are consistent with those of previous studies of mixed emotions. Researchers have found that the mixed emotion levels of dialectical and non-dialectical thinkers differed only in the predominantly pleasant situation but not in the predominantly unpleasant situation (Hui et al., 2009; Miyamoto et al., 2010; Zheng et al., 2020).

Substantial research has shown that SP/BIS and SR/BAS are valid predictors of various forms of psychopathology (Torrubia et al., 2001; Kimbrel et al., 2007), especially SP. Studies have demonstrated that higher SP can generalize anxiety disorders (Maack et al., 2012), anxiety-depression mixed disorders (Hundt et al., 2007), obsessive-compulsive disorders (Fullana et al., 2004), and longer duration of schizophrenia (Scholten et al., 2006), while lower SP can predict unipolar depression (Hundt et al., 2007). SR is less associated with mental illness and is mainly manifested in addictive behaviors; addicts have high SR (Franken et al., 2006; Scholten et al., 2006; Pardo et al., 2007; Zisserson and Palfai, 2007). An epidemiological study showed that BIS is a vulnerability factor for anxiety and depression disorders and supports the role of BAS in drug abuse and non-comorbid alcohol diagnoses; however, there is no relationship between BAS and depression diagnoses (Johnson et al., 2003). However, to distinguish between subtypes of depressive symptoms, researchers have found that low BAS predicts anhedonic depression symptoms, but not mixed anxiety–depression symptoms (Hundt et al., 2007; Kimbrel et al., 2007). Furthermore, the BIS and BAS are functionally interdependent, with each having an antagonistic effect on the actions of the other system such that low BAS may exacerbate the effects of high BIS on anhedonic depressive symptoms (Corr, 2002). Hundt et al. (2007) showed that when life stress was low, low BAS and high BIS predicted anhedonic depression. We did not find differences in SP between the two groups, whereas SR was lower in high holistic thinkers (Figure 1A), suggesting that individuals with high holistic thinking may have a predisposition to suffer from anhedonia depression when their life circumstances are generally good (less stressful in life). This is consistent with the researcher’s theory that the impact of dialectical thinking on mental health may depend on the context of the individual’s stressors and life circumstances (Wong and Liu, 2018). The best advantage of dialectical thinking for mental health should be the tendency to “find the good in the bad” when life circumstances are bad (Spencer-Rodgers et al., 2010a,b), which can reduce the harmful psychological effects of stressful situations (Ji et al., 2004). However, many studies have shown that the most significant characteristic of people with a high degree of dialecticism is particularly inclined to “find the bad in the good” (Spencer-Rodgers et al., 2010b), which might have a detrimental effect on psychological wellbeing when an individual is not experiencing hardship (Wong and Liu, 2018). Furthermore, based on the current results, individuals with high dialectical thinking tendencies are less likely to pursue positive emotions, which may lead to a relative increase in anhedonia. Further empirical studies are needed to shed light on this issue.

The amygdala and nucleus accumbens are closely related to emotions and respond to both negative and positive signals (Monk et al., 2008). Their structural alteration has been known to be associated with psychopathology (Tottenham and Galván, 2016). Researchers have found that trait anxiety is positively correlated with the bilateral volume of the nucleus accumbens (Kühn et al., 2011). Furthermore, Günther et al. (2018) revealed that higher levels of social anxiety predict increased GM volume in the right amygdala and bilateral nucleus accumbens. Adolescents with major depression disorder (MDD) have a larger nucleus accumbens volume than healthy controls (Lee et al., 2020). A meta-analysis of amygdala volume in mood disorders showed a trend toward increased left amygdala volume in adults with bipolar disorder. In addition, the left amygdala volume was larger in unipolar inpatients than in controls, whereas there were no significant changes in amygdala volume in unipolar outpatients (Hamilton et al., 2008). The effects of the amygdala in patients with MDD are unclear. The largest MDD study did not detect differences in the amygdala, nucleus accumbens, and lower hippocampal volumes (Schmaal et al., 2020). Our work suggests that the bilateral nucleus accumbens and right amygdala are smaller in individuals with high holistic thinking. Moreover, a holistic thinking tendency can negatively predict the volume of the left nucleus accumbens and right amygdala. Based on the neural results, we may be able to state that individuals with dialectical thinking report lower subjective wellbeing; however, this does not mean that their thinking styles result in bad outcomes for mental health. In terms of the aforementioned volumetric results, individuals with a high holistic thinking style may be at lower risk of anxiety and depression. However, the brain is a complex system. Some studies have demonstrated that anhedonia is associated with activity in the nucleus accumbens (He et al., 2020; Heshmati et al., 2020). Furthermore, Wacker et al. (2009) found that anhedonia symptoms of depression were correlated with reduced nucleus accumbens volume. Therefore, individuals with a high holistic tendency with smaller nucleus accumbens volume may be at risk for anhedonia symptoms.

In addition to finding that individuals with high holistic thinking are reward-insensitive and have smaller volumes in the left nucleus accumbens and right amygdala, we also found enhanced resting-state FC between the left nucleus accumbens and bilateral amygdala in high holistic thinkers than in low holistic thinkers. Beyeler et al. (2016) demonstrated that projectors between the amygdala and nucleus accumbens preferentially encode positive valence, defined as the differential response to rewarding versus aversive stimuli. Furthermore, the optogenetic activation of bilateral amygdala terminals in the nucleus accumbens is a positive reinforcement (Namburi et al., 2015), which may facilitate approval behavior. Therefore, the resting-state FC between the amygdala and nucleus accumbens may reflect individual spontaneous responsiveness to reward-related stimuli. The resting-state FC and self-reported questionnaire results were inconsistent. There are several possible reasons for this finding. First, we considered the questionnaire and resting-state brain activity results to be independent of each other and found no significant correlation between SR/SP and resting-state FC (Supplementary Table 4). Affect valuation theory proposes that how people want to feel (“ideal affect”) differs from how they actually feel (“actual affect”) and that cultural factors influence the ideal more than actual affect (Tsai et al., 2006). Resting-state FC reflects the physiological characteristics of an individual’s spontaneous brain activity (“actual affect”), while self-reported questionnaires may reflect individuals’ beliefs to some extent (“ideal affect”). Researchers have found that European Americans value high-arousal positive affect (e.g., excitement) more than Hong Kong Chinese, whereas Hong Kong Chinese value low-arousal positive affect (e.g., calm) more than European Americans (Tsai et al., 2006). Therefore, the increased FC between the nucleus accumbens and amygdala may illustrate that high holistic thinkers are sensitive to reward stimulus responses, whereas self-reported reward insensitivity may be more responsive to their belief in seeking positive emotions. Second, individuals with high holistic thinking tendency tend not to pursue high-arousal positive affect, probably because their reactivity to emotions is higher. In other words, it is because they are so responsive to emotions that something less emotionally arousing can satisfy their need for emotion, so they do not need to pursue extreme positive emotional acquisition. For example, Park et al. (2016) found that European Americans showed greater activity in the bilateral ventral striatum, including the nucleus accumbens, while viewing excited versus calm expressions, compared to the Chinese. It is noteworthy that the Chinese ventral striatum response values for both excited and clam facial expressions were negative, that is, they were relatively weaker than the resting-state activity (Park et al., 2016). Further empirical studies are required to explore this issue.

Finally, we examined the relationship between holistic thinking tendency and the volume of the nucleus accumbens and amygdala by combining them in conjunction with the RST. We found that SR partially mediated the relationship between thinking tendency and the volume of the left nucleus accumbens. Thus, SR provides some explanation for the relationship between holistic thinking tendency and nucleus accumbens volume. People with a high holistic tendency tend to avoid extreme emotions (both positive and negative) and pursue moderate emotions such as calm (Tsai et al., 2006). Therefore, this is manifested in SR as insensitivity to positive emotional events or a low tendency to acquire high-arousal positive affect. Thus, we believe that highly holistic thinkers have a low approach to acquiring positive emotions, which in turn leads to small volumes in their nucleus accumbens. It is worth noting that SR acts only as a partial mediator. There was a direct and significant relationship between the volume of the left nucleus accumbens and holistic thinking tendency (Table 2). Therefore, there are other variables that can explain the influence of holistic thinking tendencies on the nucleus accumbens. The nucleus accumbens is involved in responses to pleasure and aversive events and regulates approach and avoidance behavior (Levita et al., 2009). For example, the volume of the nucleus accumbens can predict anxiety symptoms. Burkhouse et al. (2020) demonstrated that greater left nucleus accumbens volume predicted greater decreases in clinician-rated anxiety symptoms before and after treatment. Dialectical thinkers show a greater extent of coping flexibility (Cheng, 2009; Spencer-Rodgers et al., 2010b), which in turn is associated with decreased state anxiety over time (Wong and Liu, 2018). This can also affect the nucleus accumbens.

No significant mediating effect was found for either bilateral amygdala. Substantial studies have demonstrated that across various species, the amygdala and nucleus accumbens respond to both negative and positive signals (Ernst et al., 2005; Monk et al., 2008). In particular, the nucleus accumbens is most consistently responsive to reward stimuli (Kelley, 2004; May et al., 2004; Schultz, 2004), while the amygdala responds most dramatically to negative stimuli (Whalen et al., 1998; Phelps et al., 2001). However, we did not find a relationship between SP (avoidance of negative emotional behavior) and the volumes of the nucleus accumbens and amygdala. This may be because SR and SP in the RST are completely independent and have different physiological underpinnings (Gray, 1987; Hundt et al., 2007). There are numerous neuronal connections between the amygdala and nucleus accumbens (Keistler et al., 2017; Piantadosi et al., 2017) that have been implicated in the formation of cue-reward associations (Namburi et al., 2015; Beyeler et al., 2016). In addition, resting-state FC results showed that FC between the bilateral amygdala and left nucleus accumbens was higher in individuals with high holistic thinking. Therefore, we believe that the relationship between thinking styles and the amygdala can be influenced by the nucleus accumbens. As assumed, we found that the relationship between holistic thinking tendency and amygdala volume was fully mediated by the nucleus accumbens (Supplementary Table 5). In addition, we examined whether the volume of the nucleus accumbens and amygdala and the FC between the bilateral amygdala and left nucleus accumbens mediated the relationship between holistic thinking tendency and SR. The results showed that only the left nucleus accumbens volume fully mediated the relationship between holistic thinking tendencies and SR (Supplementary Table 6).

## Conclusion

In conclusion, the current study revealed the manifestation of a holistic thinking style in the behavioral and neural substrates associated with emotional processing. On the one hand, the current study examined people’s pursuit to experience positive emotion through the RST and demonstrated that individuals with high holistic thinking tendency have a low pursuit of positive emotions. This finding explains why dialecticism is correlated with lower subjective wellbeing. In terms of neural substrates, holistic thinking tendency can negatively predict the volume of the left nucleus accumbens and right amygdala. Furthermore, our results show that there is increased resting-state FC between the bilateral amygdala and nucleus accumbens in high holistic thinkers. These findings form the neural basis for the emotionally experienced behavior of dialectical thinking.

## Data Availability Statement

The raw data supporting the conclusions of this article will be made available by the authors, without undue reservation.

## Ethics Statement

The studies involving human participants were reviewed and approved by Institute of Psychology, Chinese Academy of Sciences. The patients/participants provided their written informed consent to participate in this study.

## Author Contributions

H-XL designed and carried out the study, collected and analyzed the data, and wrote the manuscript. XH refined the research idea, designed the study, supervised the study, and revised the manuscript. Both authors contributed to the article and approved the submitted version.

## Conflict of Interest

The authors declare that the research was conducted in the absence of any commercial or financial relationships that could be construed as a potential conflict of interest.

## Publisher’s Note

All claims expressed in this article are solely those of the authors and do not necessarily represent those of their affiliated organizations, or those of the publisher, the editors and the reviewers. Any product that may be evaluated in this article, or claim that may be made by its manufacturer, is not guaranteed or endorsed by the publisher.
