# Supplementary material for: Dialectical Thinking Is Linked With Smaller Left Nucleus Accumbens and Right Amygdala
Source: Front Psychol. 2022 Feb 10;13:760489. doi: 10.3389/fpsyg.2022.760489 (PMC8866571; doi:10.3389/fpsyg.2022.760489)

**TABLE 1 |** Demographic information (n = 39).

| Variable | Mean | SD |
| --- | --- | --- |
| Age | 21.18 | 2.14 |
| Education | 2.15 | 0.43 |
| Average annual family income | 2.56 | 1.23 |
| Assessment of the socio-economic status of the family | 4.47 | 1.61 |

Education: 1. Senior high school and below; 2. Undergraduate; 3. Masters; 4. Doctor; 5. Postdoctoral; 6. Other. Average annual family income: 1. 50,000 and below; 2. 50,000 - 100,000; 3. 100,000 - 200,000; 4. 200,000 - 400,000; 5. 400,000 - 800,000; 6. 800,000 - 200,0000; 7. Over 2 million; Assessment of the socio-economic status of the family: 1 to 10.

**TABLE 2 |** The tests of normality.

|  | Kolmogorov-Smirnova | | | Shapiro-Wilk | | |
| --- | --- | --- | --- | --- | --- | --- |
|  | Statistic | df | p | Statistic | df | p |
| Holistic thinking tendency (HT) | 0.176 | 39 | 0.004 | 0.907 | 39 | 0.004 |
| reward sensitivity (SR) | 0.142 | 39 | 0.047 | 0.919 | 39 | 0.008 |
| punishment sensitivity (SP) | 0.125 | 39 | 0.129 | 0.943 | 39 | 0.047 |
| Left Nucleus Accumbens (LNAcc) | 0.089 | 39 | .200* | 0.972 | 39 | 0.431 |
| Right Nucleus Accumbens (RNAcc) | 0.085 | 39 | .200* | 0.988 | 39 | 0.954 |
| Left Amygdala (LAmy) | 0.083 | 39 | .200* | 0.972 | 39 | 0.436 |
| Right Amygdala (RAmy) | 0.062 | 39 | .200* | 0.981 | 39 | 0.73 |

* This is a lower bound of the true significance.

**TABLE 3 |** The correlation coefficients between the volume of the nucleus accumbens and amygdala and their FC (n = 39).

|  | LNAcc | RNAcc | LAmy | RAmy |
| --- | --- | --- | --- | --- |
| LNAcc_ RNAcc | 0.03 | 0.17 | 0.31 | 0.25 |
| LNAcc_ LAmy | -0.05 | -0.08 | 0.23 | 0.21 |
| LNAcc_ RAmy | 0.09 | -0.04 | 0.24 | 0.23 |
| RNAcc_ LAmy | 0.14 | -0.02 | 0.21 | 0.30 |
| RNAcc_ RAmy | 0.25 | -0.01 | 0.22 | 0.35* |
| LAmy_ RAmy | 0.24 | 0.00^b^ | -0.05 | 0.13 |

LNAcc: left nucleus accumbens, RNAcc: right nucleus accumbens, LAmy: left amygdala, RAmy: right amygdala.Two-tailed. * p < 0.05. b: 0.003.

**TABLE 4 |** The correlation of SR and SP with resting-state FC in the nucleus accumbens and amygdala, respectively. (n = 39).

|  | SR^#^ | | SP^#^ | |
| --- | --- | --- | --- | --- |
|  | Spearman’s r | p | Spearman’s r | p |
| LNAcc_ RNAcc | 0.10 | 0.53 | -0.09 | 0.61 |
| LNAcc_ LAmy | -0.02 | 0.91 | 0.06 | 0.73 |
| LNAcc_ RAmy | 0.07 | 0.69 | 0.02 | 0.91 |
| RNAcc_ LAmy | 0.01 | 0.96 | 0.29 | 0.07 |
| RNAcc_ RAmy | 0.03 | 0.85 | 0.26 | 0.11 |
| LAmy_ RAmy | 0.22 | 0.18 | 0.14 | 0.39 |

#: Non-normal distribution. LNAcc: left nucleus accumbens, RNAcc: right nucleus accumbens, LAmy: left amygdala, RAmy: right amygdala. Two-tailed.

**TABLE 5 |** Statistic on indirect and direct effects (X= holistic thinking tendency, n = 39).

| Path | Effect | Boot SE | Boot LL CI 95% | Boot UL CI 95% | Effect | Boot SE | Boot LL CI 95% | Boot UL CI 95% |
| --- | --- | --- | --- | --- | --- | --- | --- | --- |
|  | Left_ Amygdala | | | | Right_ Amygdala | | | |
| Mediator: Left_ Nucleus Accumbens | | | | | | | | |
| Indirect effect | -236.66 | 102.15 | -497.34 | -75.10 | -229.69 | 95.81 | -467.85 | -76.09 |
| Direct effect | 84.67 | 177.14 | -274.60 | 443.94 | -57.10 | 166.02 | -393.82 | -279.61 |
|  | | | | | | | | |
| Mediator: Right_ Nucleus Accumbens | | | | | | | | |
| Indirect effect | -241.81 | 90.28 | -465.74 | -97.57 | -234.74 | 93.93 | -451.91 | -82.65 |
| Direct effect | 89.81 | 163.60 | -241.98 | 421.61 | -52.04 | 152.80 | -361.95 | 257.86 |

The PROCESS model 4 was used. SE = standard error, CI = confidence interval, LL = lower limit, UL = upper limit.

**TABLE 6 |** Statistic on indirect and direct effects (X= holistic thinking tendency, Y= reward sensitivity n = 39).

| Path | Effect | Boot SE | Boot LL CI 95% | Boot UL CI 95% | Effect | Boot SE | Boot LL CI 95% | Boot UL CI 95% |
| --- | --- | --- | --- | --- | --- | --- | --- | --- |
| Mediator: | Left_ Nucleus Accumbens | | | | Right_ Nucleus Accumbens | | | |
| Indirect effect | -2.14 | 1.26 | -5.68 | -0.27 | -1.50 | 1.11 | -3.98 | 0.35 |
| Direct effect | -1.45 | 2.18 | -5.87 | 2.96 | -2.10 | 2.14 | -6.43 | -2.24 |
|  | | | | | | | | |
| Mediator: | Left_ Amygdala | | | | Right_ Amygdala | | | |
| Indirect effect | -0.12 | 0.59 | -2.22 | 0.51 | -0.13 | 0.76 | -2.03 | 1.21 |
| Direct effect | -3.47 | 2.00 | -7.52 | 0.57 | -3.47 | 2.07 | -7.65 | 0.72 |
|  |  | | | |  | | | |
| Mediator: | LNAcc_ LAmy | | | | LNAcc_ RAmy | | | |
| Indirect effect | 0.47 | 1.21 | -1.60 | 3.39 | 0.59 | 0.88 | -0.61 | 3.12 |
| Direct effect | -4.06 | 2.15 | -8.43 | 0.30 | -4.19 | 2.03 | -8.31 | -0.06 |

The PROCESS model 4 was used. SE = standard error, CI = confidence interval, LL = lower limit, UL = upper limit. LNAcc: left nucleus accumbens, LAmy: left amygdala, RAmy: right amygdala.

**TABLE 7 |** Difference in the SPSRQ questionnaire, the volume of nucleus accumbens and amygdala, and the resting-state functional connectivity between bilateral amygdala and left nucleus accumbens between groups based on median grouping.

|  | T | P | Cohen’s | 95% CI |
| --- | --- | --- | --- | --- |
| Sensitivity to Reward | -2.79 | 0.009 | 0.97 | -3.79, -0.59 |
| Sensitivity to Punishment | 1.25 | 0.222 | 0.44 | -1.11, 4.57 |
|  | | | | |
| the volume of: | | | | |
| Left Nucleus Accumbens | -3.59 | 0.001 | 1.26 | -144.48, -39.85 |
| Right Nucleus Accumbens | -2.70 | 0.011 | 0.94 | -135.79, -18.86 |
| Left Amygdala | -1.08 | 0.289 | 0.38 | -246.85, 76.12 |
| Right Amygdala | -1.83 | 0.077 | 0.64 | -281.60, 15.43 |
|  | | | | |
| Functional connectivity: | | | | |
| LNAcc_ LAmy | 2.79 | 0.009 | 0.98 | 0.06, 0.35 |
| LNAcc_ RAmy | 2.25 | 0.032 | 0.79 | 0.01, 0.28 |

LNAcc: left nucleus accumbens, LAmy: left amygdala, RAmy: right amygdala

All stimuli in the triad task


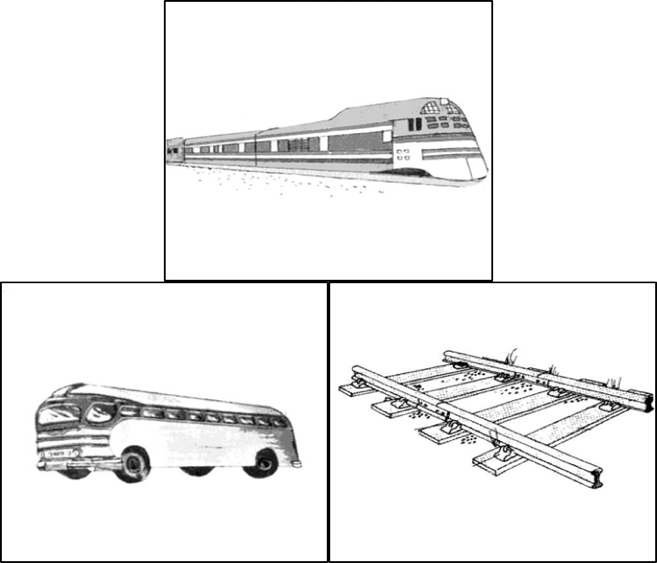


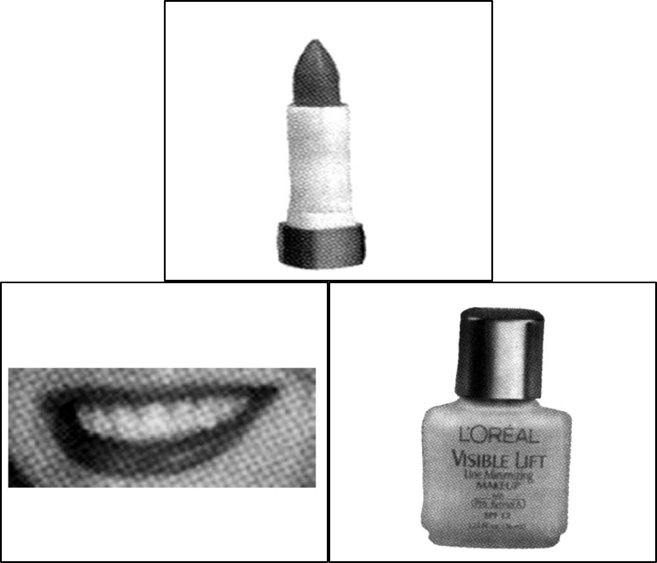


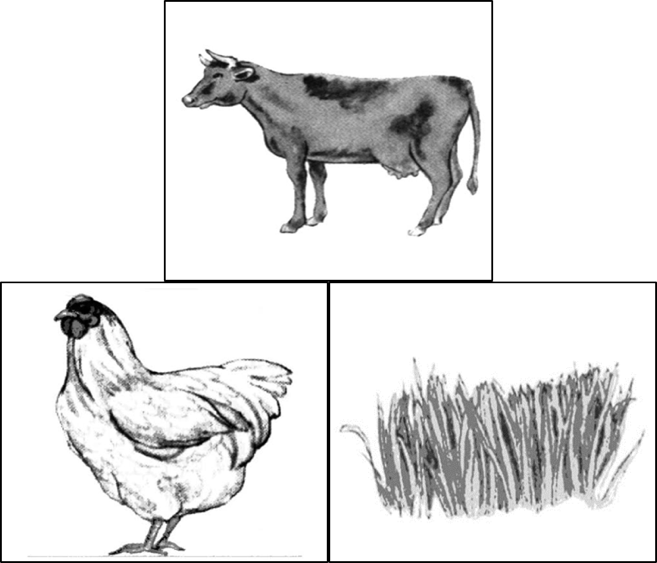


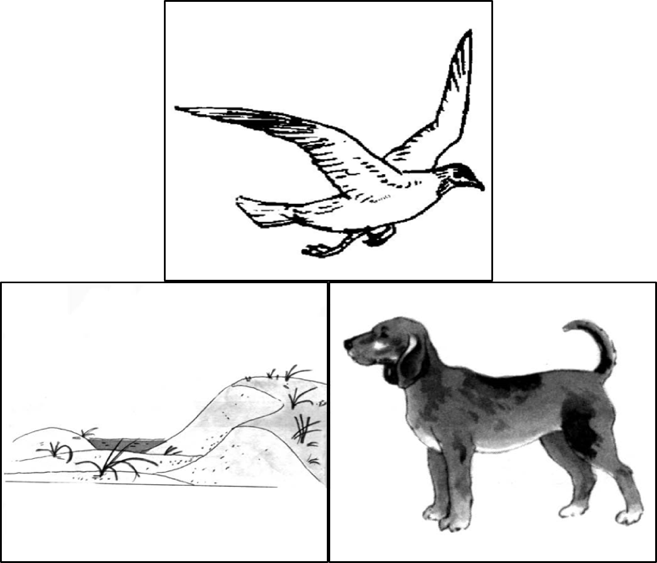


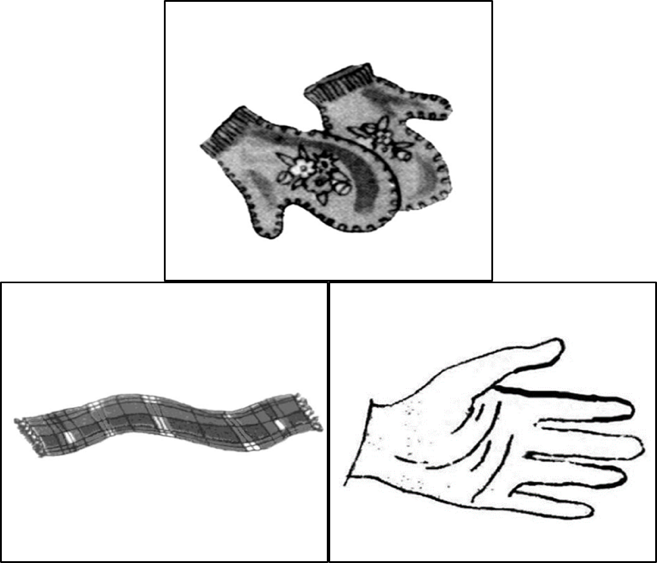


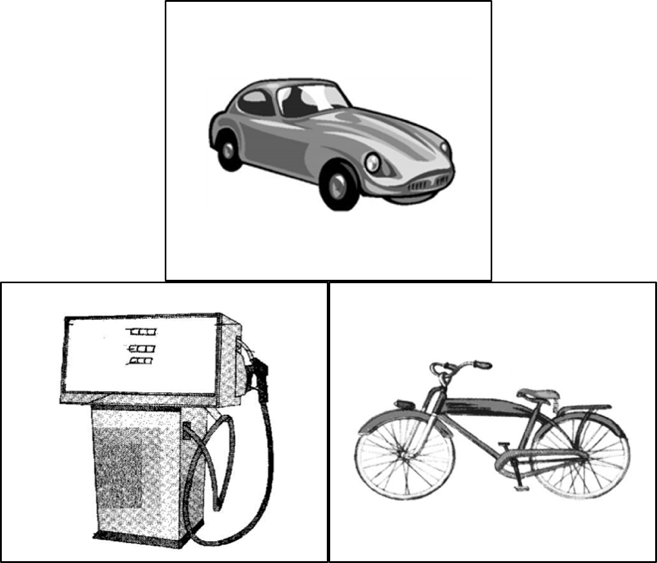


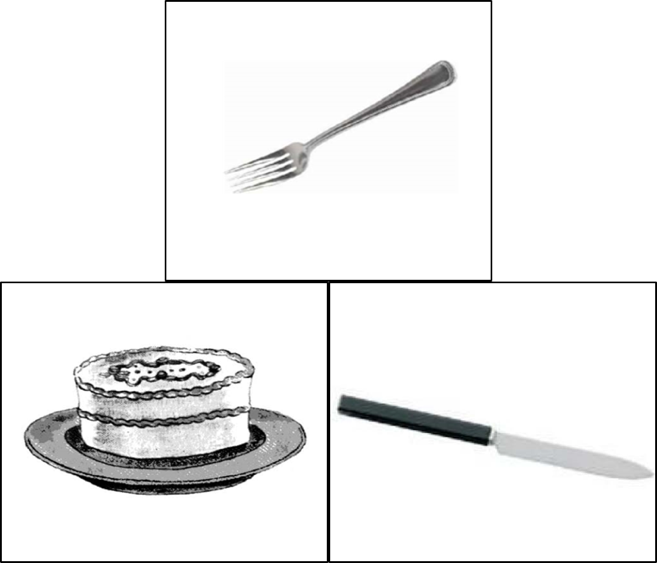


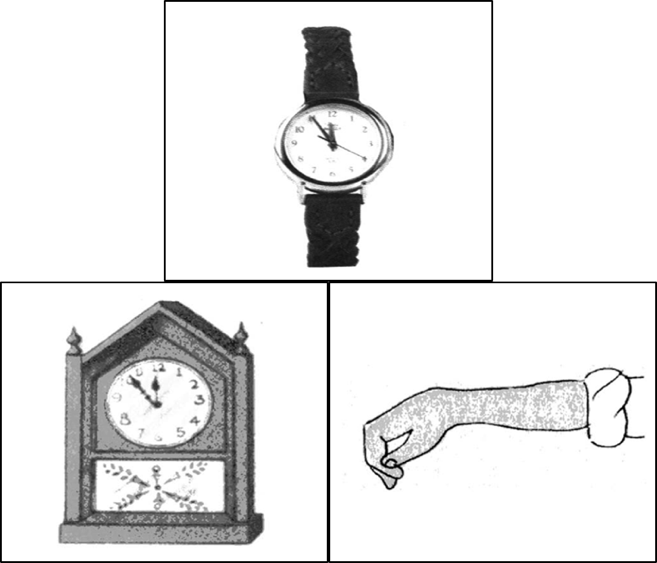


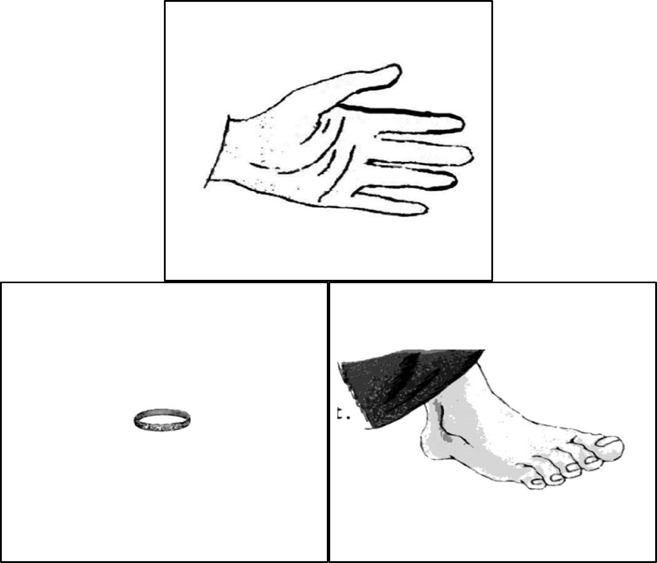


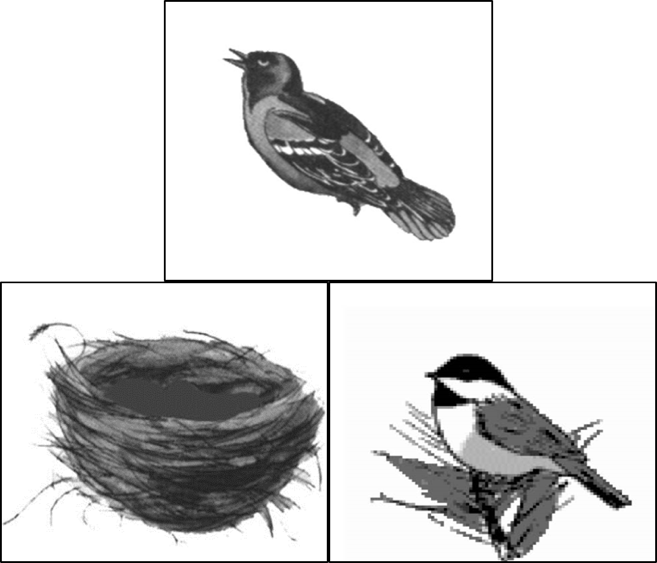


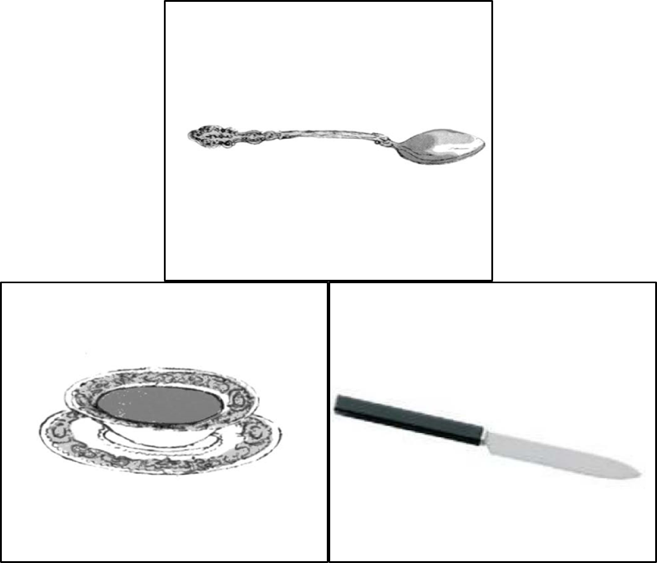


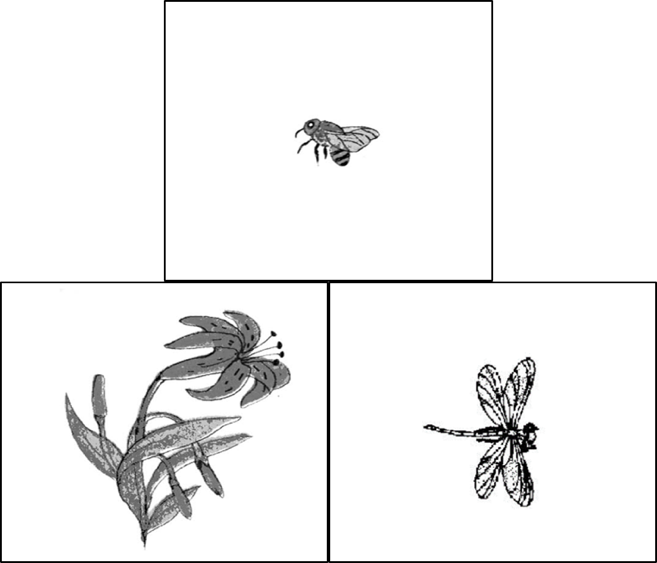


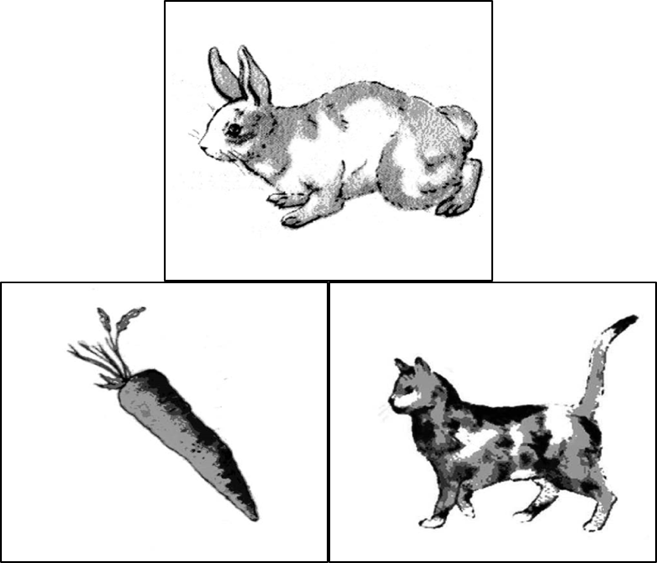


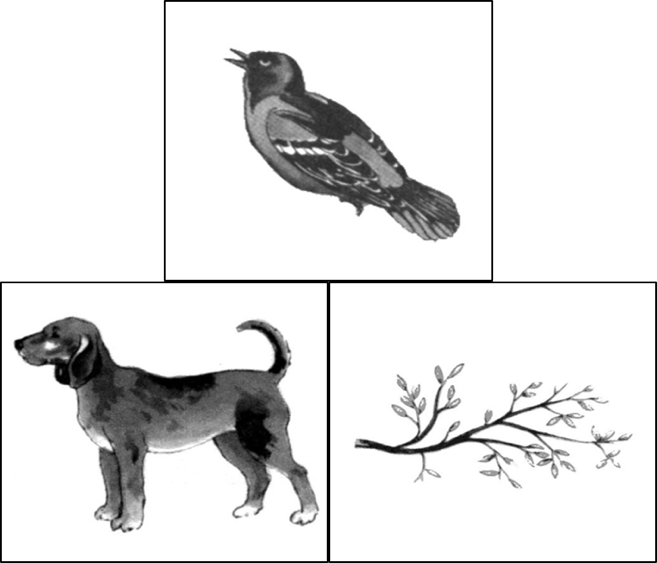

Supplement: Supplementary file 1 [file Data_Sheet_1.docx]
